# Tannic Acid Coating Augments Glioblastoma Cellular Uptake of Magnetic Nanoparticles with Antioxidant Effects

**DOI:** 10.3390/nano12081310

**Published:** 2022-04-11

**Authors:** Małgorzata Świętek, Yunn-Hwa Ma, Nian-Ping Wu, Aleksandra Paruzel, Waldemar Tokarz, Daniel Horák

**Affiliations:** 1 Institute of Macromolecular Chemistry, Czech Academy of Sciences, Heyrovského nám. 2, 162 06 Prague, Czech Republic; swietek@imc.cas.cz (M.Ś.); gawelczyk@imc.cas.cz (A.P.); 2 Department of Physiology and Pharmacology, College of Medicine, Chang Gung University, Guishan, Taoyuan 33302, Taiwan; yhma@gap.cgu.edu.tw (Y.-H.M.); ap82021@gmail.com (N.-P.W.); 3 Department of Medical Imaging and Intervention, Chang Gung Memorial Hospital, Linkou 33305, Taiwan; 4 Faculty of Physics and Applied Computer Science, AGH University of Science and Technology, Mickiewicza 30, 30-059 Krakow, Poland; tokarz@agh.edu.pl

**Keywords:** tannic acid, magnetic nanoparticles, antioxidant activity, cellular uptake

## Abstract

Coating of nanoparticles with gallates renders them antioxidant and enhances cellular internalization. In this study, (amino)silica magnetic particles modified with tannic acid (TA) and optionally with chitosan (CS) were developed, and their physicochemical properties and antioxidant activity were evaluated. The results demonstrated that the TA-modified aminosilica-coated particles, as well as the silica-coated particles with a double TA layer, exhibited high antioxidant activity, whereas the silica-coated particles with no or only a single TA layer were well-internalized by LN-229 cells. In addition, a magnet placed under the culture plates greatly increased the cellular uptake of all TA-coated magnetic nanoparticles. The coating thus had a considerable impact on nanoparticle–cell interactions and particle internalization. The TA-coated magnetic nanoparticles have great potential as intracellular carriers with preserved antioxidant activity.

## 1. Introduction

Recently observed rapid development of engineered nanoparticles creates an opportunity for their application in multiple fields, including biomedicine. Due to their small dimensions, the nanoparticles can interact with biological species on the level inaccessible for microscale materials. Such a benefit is widely exploited particularly in modern diagnostic techniques and drug delivery systems [1,2,3]. Targeted drug delivery is an important topic, especially for cancer therapies, as it can minimize overall toxicity of chemotherapeutic agents. Hence, the ability of nanoparticles to penetrate cells is a critical issue, depending on the interactions occurring at the particle–cell membrane interface. The interactions are greatly affected by the physicochemical characteristics and concentrations of nanoparticles, type of cells lines, incubation time, etc. [4]. The shape, surface charge, and coating not only play a decisive role in the mechanisms of particle internalization, but they also determine the following intracellular pathways. In general, the larger particles are taken up via phagocytosis, while the engulfment of smaller particles is mediated by membrane proteins, such as clathrin and caveolae. The presence of nanoparticles inside the cells can modify their functions both in a direct and indirect manner, influencing the biological activity by disordering cytoskeletal structures [4]. Notably, the disruption of mechanobiological behavior of cancer cells can impede tumor progression [5].

Glioblastoma multiforme, an aggressive tumor invading the brain tissue and spinal cord, is one of the most challenging cancers in terms of therapy [6]. Its fast progression together with limited therapeutic options make the 5-year survival rate of these patients among the lowest [7]. Currently, there is only one cytotoxic agent to treat glioblastoma, temozolomide. However, its application is restricted by the lack of specificity and overall toxicity resulting in adverse effects, such as nausea, vomiting, constipation, loss of appetite, and alopecia as well as increasing chemoresistance of cancer cells in response to the treatment [8]. For this reason, the development of new treatment strategies with enhanced efficacy of chemotherapy is an important issue. Depending on the stage of cancer development, approaches based on scavenging or producing radical oxygen species (ROS) have been investigated to increase the therapeutic efficacy [9,10]. ROS include various highly reactive non-radical and radical forms of oxygen. They can be generated by physiological endogenous processes in the cells including mitochondrial oxidative metabolism and can be affected by exogenous factors, such as xenobiotics, viruses, and bacteria [11]. The overbalance between production and effective ROS scavenging leads to oxidative stress, involving lipid peroxidation, damage to nucleic acids and proteins, etc. Prolonged oxidative stress causes inflammation accompanied with the development of many disorders, such as cancer, diabetes, atherosclerosis, and neurodegenerative diseases [12]. 

To combat excessive ROS formation and maintain homeostasis, organisms develop defense mechanisms involving enzymes and antioxidants. The role of antioxidants is not limited only to capturing and neutralizing ROS, but they also participate in repairing processes, enabling removal of damaged biomolecules that could negatively affect the cell metabolism [13]. Beside the endogenous antioxidants, the exogenous ones delivered mainly in food, represented by a vast group of phenolic compounds being secondary metabolites of plants, also contribute to uphold the redox balance. For this reason, naturally derived phenols play a significant role in cancer prophylaxis. On the other hand, phenolic compounds can act under specific conditions as prooxidants, contributing to the production of ROS [14]. In that case, phenolic compounds can be used as adjuvant treatment options, increasing the therapeutic outcome of cytotoxic agents. 

According to structure, the phenolic compounds can be classified into several groups, differing in basic skeleton unit. The number and type of functional groups, i.e., hydroxyls or carboxyls on the aromatic ring, determines the antioxidant capacity of phenolic compounds, which also depends on the stability of produced radicals. In general, the molecules containing multiple aromatic rings possess higher antioxidant activity than the simple phenolics [15]. Tannic acid (TA) belongs to the group of polyphenols called tannins. They have a complicated structure contributing to strong antioxidant properties, are water-soluble, and are able to form stable complexes with proteins and sugars at pH 3.5–7. Beside the antioxidant effects, TA exhibits antibacterial, anti-inflammatory, and antiviral properties, which make it an ideal candidate for various biomedical applications. To date, TA has been used in novel nanoformulations for enhanced delivery of chemotherapeutics [16,17], treatment of viral infections [18], and preparation of hydrogels for wound dressing and tissue engineering and regeneration [19,20,21,22]. Moreover, TA-functionalized magnetic nanoparticles effectively isolated circulating tumor cells and were used for immobilization of enzymes to preserve their activity [23,24]. 

Magnetic nanoparticles, especially the iron-oxide-based ones, are widely recognized for their high potential in various biomedical applications due to their biocompatibility and possibility of remote control using an external magnetic field. Previous studies indicated that polyphenolic compounds, such as tea catechins, may readily adsorb on the surface of magnetic nanoparticles in the culture medium, allowing formation of antioxidant nanocomposites; augmented cellular uptake by glioblastoma cells was observed with a synergistic effect of a magnetic field [25]. Gallate derivatives also exhibited similar effects when administered directly in the culture medium [26]. In addition, magnetic nanoparticles modified with various phenolic compounds, such as curcumin or quercetin, increased the response of pancreatic cancer cells to gemcitabine and prevented memory disjunction mediated by oxidative stress in diabetic rats [27,28]. Our previous study also demonstrated that the maghemite nanoparticles modified with a simple phenolic compound, e.g., gallic acid, enhanced nanoparticle uptake, effectively scavenged ROS, and thus reduced oxidative stress in cells [29]. 

**Figure 1:**
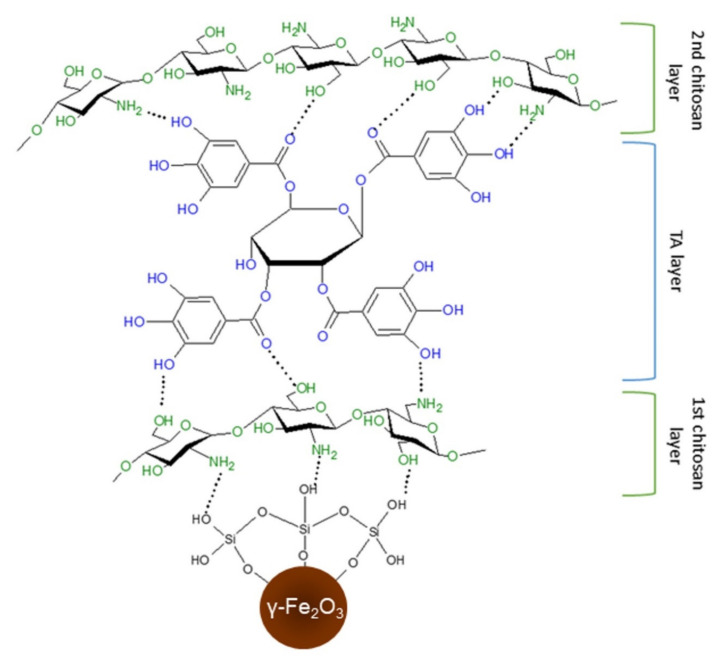
Schematic representation of interactions between TA and chitosan layers.

Although TA could increase anticancer drug uptake by tumor cells in culture, it is not clear whether this enhancement was induced by its interaction with the cells or a particular drug [16,30]. Therefore, the aim of this work was to design and prepare magnetic nanoparticles with enhanced cellular uptake and antioxidant properties, in which TA would serve as an active biocompatible molecule. To meet this target, maghemite nanoparticles were first coated with aminosilica or silica and then modified with TA or chitosan and TA using a layer-by-layer technique. Chitosan is a polysaccharide known for its biocompatibility, biodegradability, and antimicrobial and antitumor properties that make it convenient for numerous biomedical applications [31]. Amino groups of chitosan are responsible for its positive charge and capability to form complexes with hydroxy and carboxyl groups of TA; moreover, the amino groups of CS can interact with negatively charged silanol groups of silica. Furthermore, the TA can physically crosslink two chitosan layers via supramolecular interactions [32] (Figure 1). The effect of the type of silica coating, the concentration of TA, and the number of TA–chitosan layers on the ROS scavenging ability of nanoparticles and response of LN-229 glioblastoma cells was investigated.

## 2. Experimental

### 2.1. Materials 

Highly viscous chitosan (CS) from crab shells, tannic acid (TA), tetraethyl orthosilicate (TEOS), (3-aminopropyl)triethoxysilane (APTES), iron(II) chloride tetrahydrate, iron(III) chloride hexahydrate, IGEPAL^®^ CO-520 (branched poly(oxyethylene) nonylphenyl ether), ammonium persulfate, and potassium thiocyanate were purchased from Sigma-Aldrich (St. Louis, MO, USA). Dichloromethane (DCM), ethanol, 70% oleic acid (OA), glacial acetic acid, and hexane were obtained from Lach-Ner (Neratovice, Czech Republic). Dulbecco’s modified Eagle’s medium (DMEM), 0.5% trypsin-EDTA, and antibiotic-antimycotic solution were purchased from Invitrogen (Carlsbad, CA, USA). Fetal bovine serum (FBS) was from Hyclone (Logan, UT, USA). All reagents were used as delivered, without additional purification. Ultra-high purity water used for synthesis and modification of magnetic nanoparticles was produced by a Milli IQ 7000 system (Merck Millipore; Burlington, MA, USA). 

### 2.2. Synthesis of γ-Fe_2_O_3_ Nanoparticles and Their Modification with TEOS and APTES 

The magnetite (Fe_3_O_4_) nanoparticles were synthesized by the coprecipitation from aqueous FeCl_2_ and FeCl_3_ solutions, which was followed by oxidation to maghemite (γ-Fe_2_O_3_) according to the previous report [29], with slight modifications. The particles were coated by silica via a reverse microemulsion method using IGEPAL^®^ CO-520 as stabilizer [33]. Before the silanization, γ-Fe_2_O_3_ nanoparticles were transferred from aqueous to organic phase using OA as a stabilizer. At first, the γ-Fe_2_O_3_ dispersion was added to the supernatant left after the coprecipitation (two-to-one volume ratio), and then the mixture was gently poured into 2 wt.% solution of OA in DCM. Resulting OA-stabilized particles (γ-Fe_2_O_3_@OA) were washed with DCM (3 × 10 mL each) and hexane (2 × 10 mL) and finally redispersed in hexane with sonication for 10 min using a Bandelin ultrasonic homogenizer (Berlin, Germany; amplitude 10%) to reach a concentration of 3 mg/mL. 25% Ammonia solution (0.54 mL), IGEPAL^®^ CO-520 (3 mL), and TEOS (50 µL) were added under repeated homogenization for 20 min and/or stirring (800 rpm); the reaction continued at room temperature (RT) for 16 h. Resulting silica-coated nanoparticles (γ-Fe_2_O_3_@SiO_2_) were washed with ethanol, an ethanol/water solution of decreasing alcohol-to-water ratio, and water. Optionally, the γ-Fe_2_O_3_@SiO_2_ particles (60 mg) were amino-functionalized. They were dispersed in water/ethanol mixture (1:1 *v*/*v*), APTES (100 µL) was added, and the mixture was stirred (800 rpm) at 70 °C for 24 h under inert atmosphere. The resulting aminosilica-coated particles (γ-Fe_2_O_3_@SiO_2_-NH_2_) were washed with water (2 × 6 mL each) and redispersed there under sonication (10% amplitude) for 5 min. 

### 2.3. Modification of γ-Fe_2_O_3_@SiO_2_ Nanoparticles with Chitosan and Tannic Acid 

To introduce TA on the γ-Fe_2_O_3_@SiO_2_ particles, a layer-by-layer approach was used. Water-soluble low-molecular-weight chitosan (CS; *M*_n_ = 7 kDa), serving as an interlayer between the iron oxide nanoparticles and TA, was prepared by degradation of highly viscous polymer with H_2_O_2_/ascorbic acid as described previously [29]. In a typical experiment, CS (6 mg) was dissolved in water (5 mL) supplemented with 2% glacial acetic acid (0.1 mL). Separately, the γ-Fe_2_O_3_@SiO_2_ particles (10 mg) were dispersed in water (5 mL) with sonication for 5 min and added to the CS solution. The mixture was vigorously vortexed (IKA; Staufen, Germany) at RT for 1 h. The CS-modified nanoparticles (γ-Fe_2_O_3_@SiO_2_-CS) were subsequently washed with water (2 × 3 mL each) using redispersion, homogenization, and magnetic separation. To incorporate TA (being also a crosslinker of chitosan) on the γ-Fe_2_O_3_@SiO_2_-CS particles, 10 mM TA solution (0.2 or 1 mL) was diluted to 5 mL volume and vortexed with the particle dispersion for 30 min. The resulting γ-Fe_2_O_3_@SiO_2_-CS-TA02 or γ-Fe_2_O_3_@SiO_2_-CS-TA10 particles (the number in the acronym corresponds to the volume of TA solution used) were washed and redispersed in water. Optionally, the modification of γ-Fe_2_O_3_@SiO_2_-CS-TA10 particles with CS and TA was repeated with the aim of introducing additional CS and TA layers. The particles coated with double CS layers and one TA layer in between were denoted as γ-Fe_2_O_3_@SiO_2_-CS-TA-CS, while the particles with double CS and double TA layers were denoted as γ-Fe_2_O_3_@SiO_2_-CS-TA10/2. 

To prepare another set of nanoparticles, the surface of γ-Fe_2_O_3_@SiO_2_-NH_2_ particles was directly treated with TA. Analogously to the modification of γ-Fe_2_O_3_@SiO_2_-CS nanoparticles with TA, the dispersion of γ-Fe_2_O_3_@SiO_2_-NH_2_ particles (10 mg) in water (5 mL) was mixed with 0.2 or 1 mL of 10 mM TA solution, the mixture was vortexed at RT for 30 min, and the particles denoted as γ-Fe_2_O_3_@ SiO_2_-NH_2_-TA02 and γ-Fe_2_O_3_@SiO_2_-NH_2_-TA10 were washed and redispersed in water. The composition of all prepared nanoparticles is summarized in Table 1. 

### 2.4. Physicochemical Characterization of the Particles 

The γ-Fe_2_O_3_, γ-Fe_2_O_3_@SiO_2_, and γ-Fe_2_O_3_@SiO_2_-NH_2_ particles were visualized using a FEI Tecnai G2 Spirit transmission electron microscope (TEM; Brno, Czech Republic). The number-average (*D*_n_) and weight-average diameters (*D*_w_), as well as the dispersity (*Ð* = *D*_w_/*D*_n_), were calculated from the micrographs using the Atlas software (Tescan; Brno, Czech Republic). 

The particles were also characterized using dynamic light scattering (DLS), attenuated total reflection Fourier-transform infrared spectroscopy (ATR-FTIR), and thermogravimetric analysis (TGA); moreover, the magnetic properties were investigated. To determine hydrodynamic diameter (*D*_h_), polydispersity (*PD*), and ξ-potential of aqueous particle dispersions, DLS measurements were performed at 25 °C on a Zetasizer Ultra (Malvern Panalytical; Malvern, UK). Before the measurements, the particle dispersions were homogenized (10% amplitude) for 3 min and left to stand for ~10 min. ATR-FTIR spectra were measured using a Bruker IFS 55 FTIR spectrometer (Billerica, MA, USA) equipped with a mercury cadmium telluride detector and a Specac MKII Golden Gate Single Reflection ATR System (Specac; Orpington, UK) with a diamond crystal (angle of incidence 45°). The spectra were collected with a resolution of 4 cm^−1^ and 64 accumulations. TGA experiments were performed in air using a Pyris 1 thermogravimetric analyzer (PerkinElmer; Waltham, MA, USA) in the temperature range of 30 to 800 °C and heating rate of 10 °C/min. The saturation magnetization of particles was determined using a 7300 vibrating sample magnetometer (Cryotronics; Westerville, OH, USA) at 295 K. 

### 2.5. Determination of Cell-Associated Magnetic Nanoparticles (MNP_cell_) 

The LN-229 cells (Bioresource Collection and Research Center, Food Industry Research and Development Institute, Taiwan) were cultured in a 24-well culture plate until 90% confluence, which was followed by the incubation with TA-modified particles (25 or 50 µg/well) in the absence or presence of a magnet for 1–4 h. The cell-associated magnetic nanoparticles (MNP_cell_) were determined using a colorimetric method as previously described [34]. Briefly, the cells were trypsinized and subjected to 10% HCl at 55 °C for 4 h. Ammonium persulfate (1 mg/mL) was added to convert ferrous to ferric ions, which was followed by the addition of 1 M potassium thiocyanate solution, allowing formation of iron-thiocyanate complex. MNP_cell_ was determined with a VICTOR 3 multilabel plate reader (PerkinElmer; Waltham, MA, USA) at OD_490_. 

### 2.6. Antioxidant Properties of Nanoparticles 

Stable free-radical reagent DPPH was used to determine the antioxidant activity of TA-modified particles. The particle dispersion was mixed with 0.1 mM DPPH aqueous solution, and the mixture was vortexed at RT for 20 min. Then, the particles were magnetically separated, and OD_517_ was determined with a VICTOR 3 microplate reader (Per-kinElmer; Waltham, MA, USA). The antioxidant activity was calculated by comparing the decrease in DPPH absorption of particles (induced by attached TA) relative to that of control (TA). 

### 2.7. Cellular Toxicity Assay 

The toxicity of TA-modified particles toward human glioma LN-229 cells was measured using a CCK-8 kit (Sigma-Aldrich) according to the manufacturer’s instructions. Briefly, the LN-229 cells were cultured in a 96-well plate to 80–90% confluence and incubated with the particles (100 µg/mL) for 1–24 h. Then, the cells were washed with PBS and incubated with the medium containing 10% CCK-8 solution for an additional 1 h. The absorbance of each sample at 450 nm (OD_450_) was determined with a VICTOR 3 microplate reader. The percentage of cell viability was calculated as a ratio of OD of the particle-treated sample to OD of the untreated sample × 100. 

## 3. Results and Discussion 

### 3.1. γ-Fe_2_O_3_, γ-Fe_2_O_3_@SiO_2_, and γ-Fe_2_O_3_@SiO_2_-NH_2_ Nanoparticles 

**Figure 2:**
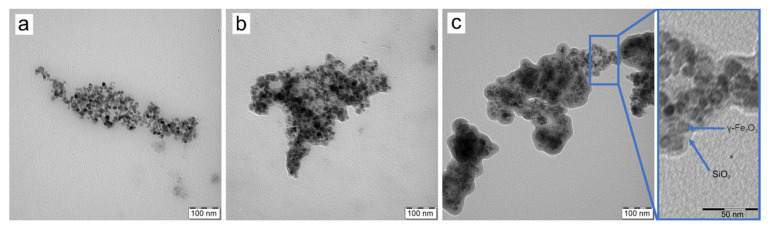
TEM micrographs of (**a**) γ-Fe_2_O_3_, (**b**) γ-Fe_2_O_3_@SiO_2_, and (**c**) γ-Fe_2_O_3_@SiO_2_-NH_2_ particles.

The developed γ-Fe_2_O_3_ nanoparticles were round shaped and possessed a moderate dispersity typical for the particles prepared by the coprecipitation method. The number-average diameter (*D*_n_) and dispersity (*Ð*) determined from the TEM micrographs were 13 nm and 1.19, respectively (Figure 2a). The hydrodynamic diameter (*D*_h_) of γ-Fe_2_O_3_ nanoparticles in water (91 nm) was seven times higher than the *D*_n_ due to the presence of the solvation layer and partial particle aggregation. However, the high value of ζ-potential (48 mV; Table 1) indicated the good colloidal stability of particles in water due to effective repulsions. 

**Figure 3:**
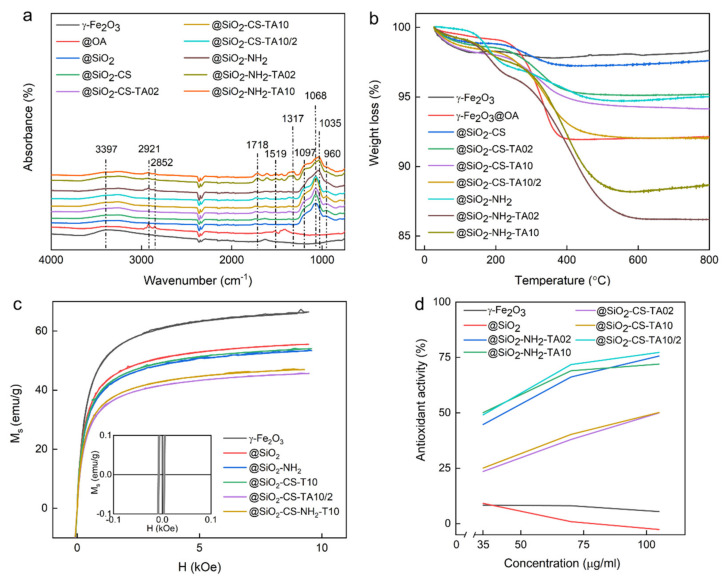
(**a**) ATR-FTIR spectra, (**b**) TGA thermograms, (**c**) dependence of saturation magnetization on magnetic field at 293 K for variously modified nanoparticles (insert shows the hysteresis curve of γ-Fe_2_O_3_ nanoparticles at low magnetic field), and (**d**) radical-scavenging activity of TA-modified γ-Fe_2_O_3_ nanoparticles determined by DPPH assay.

In the ATR-FTIR spectrum of unmodified γ-Fe_2_O_3_ nanoparticles (Figure 3a), two main peaks at 1629 and 3397 cm^−1^ assigned to the O-H scissor and stretching vibrations, respectively, corresponded to the absorbed water [35]. According to TGA, the content of water, both adsorbed and crystalline, was ~2 wt.% (Figure 3b). The saturation magnetization (*M*_s_) of unmodified magnetic nanoparticles was 67 emu/g, which corresponded to the results obtained in other studies [36,37] and indicated that magnetite was partially oxidized to maghemite (Figure 3c). The limited oxidation of magnetite to maghemite was also confirmed by the elemental analysis, which indicated content of iron between those expected for γ-Fe_2_O_3_ and Fe_3_O_4_ [38]. Moreover, the color changed from black to brownish, clearly indicating the surface oxidation of Fe_3_O_4_ nanoparticles. The small values of coercivity (0.01 Oe) and magnetic remanence (0.6 emu/g) proved that the nanoparticles were superparamagnetic with a small admixture of large ferrimagnetic particles.

Prior to the silica coating, the γ-Fe_2_O_3_ nanoparticles were transferred from aqueous to organic phase by the addition of OA. The formation of the OA layer on the particle surface resulted in the appearance of new peaks in the FTIR spectrum at 1416, 1465, 1519, 2852, and 2921 cm^−1^ (Figure 3a). The peak at 1416 cm^−1^ corresponded to the CH_3_ umbrella vibrational mode of OA [39]. The peaks at 1465 and 1519 cm^−1^ were assigned to the asymmetric and symmetric vibrations of the COOH functional group, respectively, while the peaks at 2852 and 2921 cm^−1^ were ascribed to the –CH symmetric and asymmetric vibrations of OA, respectively. The typical peak of OA at 1708 cm^−1^ (C=O stretching vibration) was not detected due to binding of carboxyl groups to iron oxide. According to the TGA of γ-Fe_2_O_3_@OA nanoparticles, the total weight loss amounted to 8 wt.%, from which 6 wt.% could be assigned to OA and ~2 wt.% was ascribed to water bound by γ-Fe_2_O_3_ nanoparticles as already mentioned above (Figure 3b).

The silica layer surrounding magnetic nanoparticles after their silanization was clearly visible on the TEM micrograph as a halo (Figure 2b); the thickness of the layer was ~5 nm. In the DLS measurements, coating of the particles with silica changed the ζ-potential from positive to negative values, and *D*_h_ increased by ~130 to 228 nm (Table 1). However, the relatively low value of polydispersity (*PD*) and the high absolute value of ζ-potential indicated that the significant enhancement of *D*_h_ was associated with the larger diameter of γ-Fe_2_O_3_@SiO_2_ nanoparticles. The negative charge of γ-Fe_2_O_3_@SiO_2_ particles originated from the deprotonation of silanol groups on the silica surface [40]. Moreover, the silica coating diminished peaks assigned to OA in the FTIR spectrum of γ-Fe_2_O_3_@SiO_2_ particles, and new peaks appeared in the range of 960 to 1370 cm^−1^ (Figure 3a). The most intensive peak at 1068 cm^−1^ was attributed to the Si-O-Si stretching vibration, while that at 960 cm^−1^ corresponded to the Si–O–H bending in silanol groups, confirming the formation of a silica layer on the particle surface [41,42,43]. The total weight loss of silica-coated particles was 4 wt.%, which was equally attributed to both water evaporation (up to 280 °C) and removal of post-synthetic residues after incomplete hydrolysis of TEOS (up to 500 °C). Compared to the bare γ-Fe_2_O_3_ particles, the saturation magnetization (*M*_s_) of silica-modified nanoparticles was lower by 12 emu/g, reaching 55 emu/g, which resulted from increased contribution of the non-magnetic silica phase on the nanoparticles. 

As a result of aminosilica functionalization, the thickness of the halo layer around the particles increased by 8 to 13 nm (Figure 2c). According to DLS, the ζ-potential was changed from negative to positive values due to the presence of amino groups on the surface of γ-Fe_2_O_3_@SiO_2_-NH_2_ particles (Table 1). Moreover, *D*_h_ increased by ~20 nm without significantly changing *PD*, confirming the formation of an aminosilica layer on the Fe_2_O_3_@SiO_2_ particles (Table 1). In the FTIR spectrum of γ-Fe_2_O_3_@SiO_2_-NH_2_ particles, the band at 1068 cm^−1^ was slightly modified, and two additional peaks at 2852 and 2921 cm^−1^ were assigned to the C–H and C–N stretching vibrations, respectively, confirming the introduction of amino groups on the silica surface (Figure 3a) [42]. According to the TGA results, the total weight loss of γ-Fe_2_O_3_@SiO_2_-NH_2_ particles up to 600 °C amounted to 5.2 wt.% (Figure 3b). This agreed with the literature, where the weight loss of amino-functionalized silica nanoparticles was induced by the removal of adsorbed water and gases (up to 130 °C) and gradual decomposition of aminopropyl groups (up to 380 °C) [43]. The introduction of an aminosilica layer also reduced the *M*_s_ to 53 emu/g. 

The purpose of coating the γ-Fe_2_O_3_ particles with silica was to create a physical boundary between the particles and the surrounding media. Both iron and iron oxide nanoparticles are prone to oxidation and reduction, resulting in the formation or scavenging of ROS; at the same time, the particle surface changes, leading to degradation, increased cytotoxicity, and unwanted loss of magnetic properties [44]. The silica coating on magnetic nanoparticles thus has a protective role, minimizing the release of Fe ions, as described in numerous studies [45,46,47,48]. Furthermore, in our study the silica and aminosilica layers provided particle stability and prevented a complex biding with TA. 

### 3.2. γ-Fe_2_O_3_@SiO_2_-CS-TA Nanoparticles

The modification of negatively charged γ-Fe_2_O_3_@SiO_2_ particles with TA firstly required the change of ζ-potential to positive values, which was achieved by coating the particles with CS. The attachment of CS to the particle surface had no impact on *D*_h_ (Table 1). The significant drop in the absolute value of ζ-potential (by 47 mV) indicated relatively low colloidal stability of the γ-Fe_2_O_3_@SiO_2_-CS particles in water due to their weak repulsions, as well as limited interactions between the hydroxyl and amino groups of silica and CS, respectively. As a result, only a small amount of CS was adsorbed on the γ-Fe_2_O_3_@SiO_2_ surface as confirmed by TGA, where the difference between the total weight loss of γ-Fe_2_O_3_@SiO_2_-CS and γ-Fe_2_O_3_ at 600 °C was only 0.7 wt.% (Figure 3b). According to the previous study [29], the main peaks in the FTIR spectrum of CS were expected at 1028, 1064, and 1552 cm^−1^; however, two peaks at lower wavenumbers were likely overlapped by the intensive peak of silica, and the peak at 1552 cm^−1^ was not identified. The lack of signals from CS in the ATR-FTIR spectrum of γ-Fe_2_O_3_@SiO_2_-CS thus also indicated rather low content of CS attached to the particle surface. 

To modify the γ-Fe_2_O_3_@SiO_2_-CS with TA, its two concentrations were used; 0.2 and 1 mM TA solution was added to the particle dispersion during the synthesis, resulting in the formation of γ-Fe_2_O_3_@SiO_2_-CS-TA02 and γ-Fe_2_O_3_@SiO_2_-CS-TA10 nanoparticles, respectively. Here, the attachment of TA reduced both *D*_h_ (by 8 nm) and ζ-potential (to −2 mV) compared to the values of γ-Fe_2_O_3_@SiO_2_-CS (Table 1). The negative ζ-potential of γ-Fe_2_O_3_@SiO_2_-CS-TA02 and γ-Fe_2_O_3_@SiO_2_-CS-TA10 nanoparticles resulted from the presence of carboxyl groups in TA. Compared to the FTIR spectrum of γ-Fe_2_O_3_@SiO_2_-CS particles, their modification with TA led to the appearance of a new peak at 1317 cm^−1^ and increased intensity of peaks at 1365 and 1718 cm^−1^ in the spectrum of γ-Fe_2_O_3_@SiO_2_-CS-TA (Figure 3a). The peak at 1718 cm^−1^ corresponded to the C=O vibration of TA, while the band at 1317 cm^−1^ was assigned to stretching (C_ar_–C_ar_ and C_ar_–OC) and bending in plane (C_ar_–OH) vibrations associated with the substituted benzene ring [49]. Based on the TGA results, the amount of TA on particles was concentration-dependent, amounting to ~2.2 and 3.1 wt.% for γ-Fe_2_O_3_@SiO_2_-CS-TA02 and γ-Fe_2_O_3_@SiO_2_-CS-TA10, respectively (Figure 3b). The minor contribution of TA to the total mass of nanoparticles was also reflected in small difference (1 emu/g) between the *M*_s_ of γ-Fe_2_O_3_@SiO_2_ and that of γ-Fe_2_O_3_@SiO_2_-CS-TA10. 

To increase the amount of TA on particles, additional CS and TA layers were introduced on the γ-Fe_2_O_3_@SiO_2_-CS-TA10 nanoparticles. The introduction of the second chitosan layer was accompanied with increasing *D*_h_ (by 19 to 237 nm). Similar to the γ-Fe_2_O_3_@SiO_2_-CS nanoparticles, the γ-Fe_2_O_3_@SiO_2_-CS-TA-CS ones had a ζ-potential of 7 mV. No significant changes in the FTIR spectrum of γ-Fe_2_O_3_@SiO_2_-CS-TA-CS were observed compared to that of γ-Fe_2_O_3_@SiO_2_-CS-TA10 particles. 

The addition of the second TA layer to γ-Fe_2_O_3_@SiO_2_-CS-TA particles slightly increased *D*_h_ (by 11 to 248 nm) and substantially decreased ζ-potential (by 27 to −20 mV), indicating the stabilizing effect of TA (Table 1). Such a value of the ζ-potential agreed with those published earlier for TA-functionalized magnetic nanoparticles [23]. In the FTIR spectrum of γ-Fe_2_O_3_@SiO_2_-CS-TA10/2 particles, the enhanced amount of TA antioxidant was documented by a slight increase in the intensity of peaks at 1365 and 1619 cm^−1^ and the appearance of a new band at 1317 cm^−1^ (Figure 3a). According to the TGA, the contribution of the second CS and TA layer to the total particle mass amounted to 2.1 wt.%, and thus the total content of TA on γ-Fe_2_O_3_@SiO_2_-CS-TA10/2 particles reached ~5 wt.% (Figure 3b). The *M*_s_ of nanoparticles with the double CS-TA layer was the lowest from all produced particles, reaching 47 emu/g. 

According to our previous study, the low-molecular-weight chitosan used for the modification of γ-Fe_2_O_3_@SiO_2_ and γ-Fe_2_O_3_@SiO_2_-CS-TA10 particles showed negligible antioxidant activity expressed as gallic acid equivalent [29]. Consequently, the γ-Fe_2_O_3_@SiO_2_-CS and γ-Fe_2_O_3_@SiO_2_-CS-TA-CS particles were excluded from the evaluation of antioxidant properties. Here, the negatively charged γ-Fe_2_O_3_@SiO_2_ nanoparticles served as a control, as the γ-Fe_2_O_3_@SiO_2_-CS-TA and γ-Fe_2_O_3_@SiO_2_-NH_2_-TA particles also possessed negative charge. The negative charge of silica-coated nanoparticles resulting from the presence of silanol groups enabled coating with CS using the layer-by-layer technique. This method has been used to deposit CS not only on the nanoparticles [50] but also on silicon chips [51] and bioactive glass containing silica particles [52]. Moreover, the possible interactions between CS and TA were investigated using gallic acid (monomer of tannic acid) as a model. It was found that the ability of different hydroxyl groups in TA to interact depended on their positions in the aromatic ring [53]. In the case of CS, the protonated amino groups were involved in electrostatic interactions. Additionally, the CS-TA complex was stabilized by hydrogen bonds and van der Waals interactions, enabling TA to act as a physical cross-linker for the particles containing two chitosan layers [54,55,56,57].

To determine whether the pattern of TA-modified nanoparticle cellular uptake was associated with antioxidant activities, the scavenger effects of particles were determined using DPPH assay (Figure 3d). The antioxidant activities of γ-Fe_2_O_3_, γ-Fe_2_O_3_@SiO_2_, and TA-modified nanoparticles were found to be concentration-dependent. Antioxidant activities of nanoparticles with one TA layer, including γ-Fe_2_O_3_@SiO_2_-CS-TA02 and γ-Fe_2_O_3_@SiO_2_-CS-TA10, were lower (25 and 45% at 35 and 100 µg/mL, respectively) than those for double TA-layered γ-Fe_2_O_3_@SiO_2_-CS-TA10/2 particles (50 and 77% at 35 and 100 µg/mL, respectively). In contrast, the γ-Fe_2_O_3_ and γ-Fe_2_O_3_@SiO_2_ particles in all concentrations studied exhibited no scavenging effect against DPPH. This indicated that the number of TA layers significantly increased the antioxidant properties of particles. This agreed with the literature, where the antibacterial properties of the CS-TA system depended on the number of deposited TA layers [58]. 

### 3.3. γ-Fe_2_O_3_@SiO_2_-NH_2_-TA Nanoparticles 

Similar to the modification of γ-Fe_2_O_3_@SiO_2_-CS nanoparticles, the γ-Fe_2_O_3_@SiO_2_-NH_2_ particles were also treated with two concentrations of TA (0.2 and 1 mM) to yield γ-Fe_2_O_3_@SiO_2_-NH_2_-TA02 and γ-Fe_2_O_3_@SiO_2_-NH_2_-TA10, respectively. Here, TA was expected to interact directly with the amino groups of γ-Fe_2_O_3_@SiO_2_-NH_2_ particles. The γ-Fe_2_O_3_@SiO_2_-NH_2_-TA02 particles had almost the same *D*_h_ as the γ-Fe_2_O_3_@SiO_2_-NH_2_. In contrast, the *D*_h_ of γ-Fe_2_O_3_@SiO_2_-NH_2_-TA10 particles was smaller by 20 nm compared to that of γ-Fe_2_O_3_@SiO_2_-NH_2_ particles probably due to the improved stabilization from the TA. As expected, both types of particles were negatively charged with the ζ-potential <−20 mV. The lower values of both *D*_h_ and ζ-potential of γ-Fe_2_O_3_@SiO_2_-NH_2_-TA10 particles indicated their slightly higher colloidal stability in water compared to those of γ-Fe_2_O_3_@SiO_2_-NH_2_-TA02. The FTIR spectra of γ-Fe_2_O_3_@SiO_2_-NH_2_-TA02 and γ-Fe_2_O_3_@SiO_2_-NH_2_-TA10 particles were identical in terms of peak intensities and positions (Figure 3a). Compared to the spectrum of γ-Fe_2_O_3_@SiO_2_-NH_2_ particles, new peaks were observed in the spectrum of γ-Fe_2_O_3_@SiO_2_-NH_2_-TA particles in the range of 1097 to 1718 cm^−1^ that corresponded to C-O and CH_2_ vibrations in aromatic rings and C = O stretching vibrations [59]. According to the TGA results, the TA content in γ-Fe_2_O_3_@SiO_2_-NH_2_-TA02 and γ-Fe_2_O_3_@SiO_2_-NH_2_-TA10 particles reached 8.7 and 6.4 wt.%, respectively. The *M*_s_ of γ-Fe_2_O_3_@SiO_2_-NH_2_-TA10 was 46 emu/g, which was almost an identical value to that for particles with the double chitosan-TA layer. 

When considering the ability of aminosilica-coated γ-Fe_2_O_3_ nanoparticles to remove DPPH radicals, it was found that the γ-Fe_2_O_3_@SiO_2_-NH_2_-TA02 and γ-Fe_2_O_3_@SiO_2_-NH_2_-TA10 particles exhibited high antioxidant activity, only slightly lower compared to that of γ-Fe_2_O_3_@SiO_2_-CS-TA10/2. The γ-Fe_2_O_3_@SiO_2_-NH_2_-TA02 and γ-Fe_2_O_3_@SiO_2_-NH_2_-TA10 particles at concentrations of 35 and 100 µg/mL scavenged 45–50 and 72–75% of free ra-dicals, respectively. Previously, magnetic mesoporous aminosilica particles were reported as an adsorbent for removing TA from aqueous solutions in a concentration-dependent manner [60]. In another study, the amino groups of aminosilica microspheres were crosslinked with hydroxyl groups of TA via glutaraldehyde [61]. Such microspheres exhibited enhanced antibacterial activity and increased rate of wound healing. A different strategy consisted of the immobilization of TA on magnetic silica microspheres using Fe^3+^ ions [62], where TA was likely to create a complex bond with amino groups of aminosilica coating. The similar ability of the γ-Fe_2_O_3_@SiO_2_-NH_2_-TA02 and γ-Fe_2_O_3_@SiO_2_-NH_2_-TA10 particles to scavenge DPPH radicals thus indicated that the lower concentration of TA was enough to bind the majority of accessible amine groups. 

### 3.4. Uptake of Differently Coated γ-Fe_2_O_3_ Nanoparticles by LN-229 Cells 

**Figure 4:**
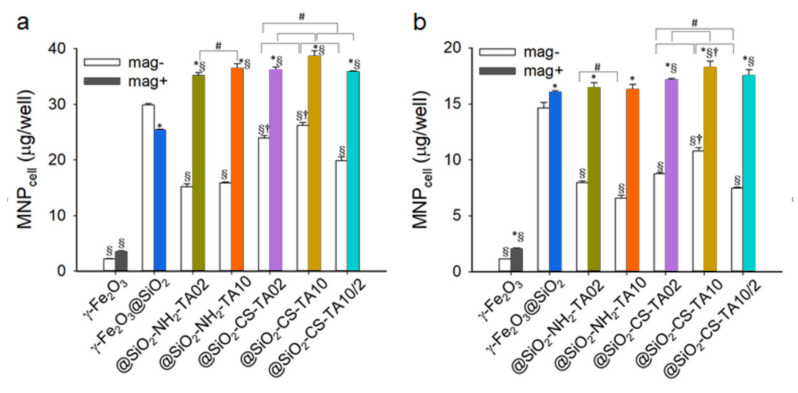
Uptake of the TA-modified γ-Fe_2_O_3_ particles by LN-229 cells. Magnetic field was applied for 5 min (mag−) and 1 h (mag+) after administration of particles at concentration of (**a**) 50 or (**b**) 25 µg/well. Values are means ± SEM (*n* = 4); the results are representative of three experiments using different batches of cells. *, *p* <0.05 relative to mag− group. §, *p* < 0.05 relative to γ-Fe_2_O_3_@SiO_2_. †, *p* < 0.05 relative to group without chitosan. #, *p* < 0.05 relative to group with different concentrations of TA.

The uptake of magnetic nanoparticles was tested on an LN-229 cell line derived from parieto-occipital glioblastoma. This cell line was selected because it is widely investigated in the literature and can serve as a model when the cellular uptake of magnetic nanoparticles with antioxidant effects is evaluated. As the cellular uptake was determined by the surface properties of particles, the composition of magnetite core surrounded by the uniform maghemite shell did not affect the cellular response; the preparation of core-shell particles is a widely recognized approach to reduce the toxicity of core particles [63]. Thus, the presence of maghemite enhanced the biocompatibility of particles. The comparison of PC3 human prostate cancer cell response to magnetite and maghemite nanoparticles revealed that the latter particles exhibited lower toxicity and higher internalization [64]. Similarly, the maghemite particles were less toxic towards RAW 264.7 murine peritoneal macrophages than the magnetite ones [65]. These studies demonstrated that polyphenolic compounds augmented uptake of nanoparticles in the human brain glioblastoma LN-229 cell line [25,26]. To determine the effect of TA coating on the internalization of magnetic particles by living cells, the quantity of cell-associated magnetic nanoparticles (MNP_cell_) was determined after the incubation of particles (50 µg/well) with LN-229 cells for 1 h (Figure 4). In the absence (mag−) and presence of a magnetic field (mag+), the silica coating enhanced the MNP_cell_ value more than 13-fold and 7-fold, respectively, compared to that without silica coating (Figure 4a). The application of a magnetic field during the incubation with TA-modified particles increased the MNP_cell_ level by 1.5 times to more than double compared to that in the absence of a magnetic field. In the mag− group, TA modification decreased the MNP_cell_ value to 12–49% of that for γ-Fe_2_O_3_@SiO_2_. However, this mo-dification increased MNP_cell_ by almost 1.5 times compared to that in the mag+ group. CS modification then increased MNP_cell_ 1.6–1.7 times compared to corresponding mag− group without chitosan. Among the particles with chitosan modification, higher concentration of TA (TA10 particles) induced higher uptake than that at lower concentration (TA02 particles). Nevertheless, the γ-Fe_2_O_3_@SiO_2_-CS-TA10/2 particles with two layers of TA exhibited a lower MNP_cell_ value than that for the γ-Fe_2_O_3_@SiO_2_-CS-TA02 or γ-Fe_2_O_3_@SiO_2_-CS-TA10 in the mag− group (Figure 4a). The results thus suggested that TA coating allowed uptake of nanoparticles in LN-229 cells via a distinct endocytosis mechanism. Such a mechanism may mediate the enhanced uptake of anticancer nanocomposites observed previously [16,30]. Moreover, the results of the DPPH test and the uptake stu-dies indicated that the nanoparticle uptake by LN-229 cells was not dependent on the antioxidant activity, which is consistent with previous findings [25]. 

To determine whether the lower concentration of nanoparticles increases their uptake by cells, the particle concentration was reduced by half (to 25 µg/well) with the same experimental design (Figure 4b). In the mag− vs. mag+ groups, the silica coating increased the MNP_cell_ values in LN-229 cells by 12.7 times vs. 7.7 times, respectively, compared to neat nanoparticles. The application of a magnetic field during the incubation with TA-modified nanoparticles increased the MNP_cell_ level 1.7–2.5 times compared to that in corresponding mag− groups. TA modification decreased MNP_cell_ to 26–55% relative to that of γ-Fe_2_O_3_@SiO_2_ in the mag− group. Compared to the nanoparticles with TA10, CS modification increased MNP_cell_ 1.6 times and 1.1 times in the mag− and mag+ groups, respectively. Similar to Figure 4a, the γ-Fe_2_O_3_@SiO_2_-CS-TA10/2 nanoparticles exhibited lower MNP_cell_ than that for γ-Fe_2_O_3_@SiO_2_-CS-TA02 or γ-Fe_2_O_3_@SiO_2_-CS-TA10 in the mag− group. The results were consistent with those in Figure 4a, suggesting that the γ-Fe_2_O_3_@SiO_2_ particles were highly effective at entering cells in the mag− group. In addition, the γ-Fe_2_O_3_@SiO_2_-CS-TA10 particles appeared to be the most effective, being internalized with a distinct endocytosis pathway. Moreover, the doubled particle concentration increased uptake of particles by LH-229 cells twice (Figure 4). 

**Figure 5:**
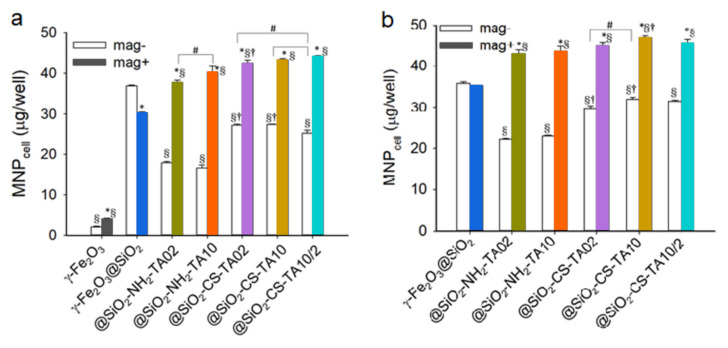
Uptake of TA-modified γ-Fe_2_O_3_ particles (50 µg/well) by LN-229 cells after incubation for (**a**) 2 and (**b**) 4 h in the presence (mag+) or absence of magnet (mag−). Values are means ± SEM (*n* = 4). *, *p* < 0.05 relative to mag− group. §, *p* < 0.05 relative to γ-Fe_2_O_3_@SiO_2_. †, *p* < 0.05 relative to group without chitosan. #, *p* < 0.05 relative to group with different concentrations of TA.

To determine the optimal incubation time for the particles, 2 and 4 h incubation periods were investigated. In the 2 h incubation experiments, all nanoparticles, except γ-Fe_2_O_3_@SiO_2_, increased the MNP_cell_ level in the presence of a magnet compared to that in the mag− group (Figure 5a). In the mag− vs. mag+ groups, the silica coating enhanced the MNP_cell_ value by 17.5 times vs. 7.4 times, respectively, compared to that obtained with particles without silica. TA modification decreased the MNP_cell_ value in LN-229 cells by 26–55% compared to that for γ-Fe_2_O_3_@SiO_2_ in the mag− group. CS modification of TA-containing nanoparticles increased MNP_cell_ 1.5–1.6 times compared to that in the corresponding mag− group with particles without CS. The γ-Fe_2_O_3_@SiO_2_-CS-TA10/2 nanoparticles exhibited a lower MNP_cell_ value than that for γ-Fe_2_O_3_@SiO_2_-CS-TA02 and γ-Fe_2_O_3_@SiO_2_-CS-TA10 in the mag− group (Figure 5a). 

With longer incubation time (4 h), the application of a magnetic field during the incubation with TA-modified nanoparticles increased the MNP_cell_ level 1.4–1.9 times compared to that in the mag− group (Figure 5b). The TA-modified particles also decreased the MNP_cell_ value to 11–37% compared to that of γ-Fe_2_O_3_@SiO_2_ in the mag− group. Nevertheless, the TA-modified nanoparticles increased the MNP_cell_ value 1.2–1.3 times compared to that for γ-Fe_2_O_3_@SiO_2_ in the mag+ group. CS- and TA-modified nanoparticles increased the MNP_cell_ value 1.3–1.4 times compared to the corresponding mag− group with particles without CS. The results indicated that similar patterns of MNP_cell_ were observed for both 2 and 4 h incubation time, suggesting that the short incubation time (1 h) was enough to differentiate the effects of coatings. 

Since the potential toxicity may impede particle internalization, the cell viability CCK-8 assay was used to access cytotoxicity of TA-modified nanoparticles. The results demonstrated that no significant cytotoxicity was observed after the incubation of particles with LN-229 cells for 1–24 h (data not shown), suggesting that cytotoxicity is unlikely to mediate effects on nanoparticle internalization. 

## 4. Conclusions

Phenolic-modified magnetic nanoparticles are of practical importance particularly for biomedical applications. In this work, we have demonstrated the successful synthesis of tannic acid-modified magnetic nanoparticles with antioxidant properties using two approaches: reaction of TA with chitosan-modified γ-Fe_2_O_3_@SiO_2_ nanoparticles and direct treatment of aminosilica-coated γ-Fe_2_O_3_ particles with TA. The particles were fully characterized by TEM, DLS, FTIR, TGA, magnetometric, and antioxidant analyses. The coating material exhibited great impact on nanoparticle–cell interaction and thus particle internalization. While the SiO_2_ modification greatly enhanced cellular uptake of nanoparticles, it has been shown to induce toxicity, such as hemolysis [66], which jeopardizes the biocompatibility of the nanoparticles and limits their clinical application in humans. The TA coating of aminosilica iron oxide particles and/or γ-Fe_2_O_3_@SiO_2_ particles with double TA/CS layers not only provided antioxidant properties but also induced a shielding effect that resulted in reduced cellular uptake. Nevertheless, coating with TA still greatly enhanced cellular uptake compared to that of γ-Fe_2_O_3_ core in the presence or absence of the magnet. These results are consistent with previous findings [29] that the immobilization of gallate derivatives on magnetic nanoparticles greatly enhanced internalization by tumor cells compared to that of γ-Fe_2_O_3_. The internalization of TA-modified particles in LN-229 cells was also substantially increased when placed in an external magnetic field as demonstrated by the determination of the quantity of cell-associated magnetic nanoparticles. Whether augmented cellular uptake associated with TA nanocomposites conveys an enhanced targeting effect in a tumor model remains to be investigated. 

## Figures and Tables

**Table 1:** Composition and DLS characterization of the magnetic nanoparticles.

Particles	Number of Layers					*D*_h_ (nm)	*PD*	ζ-Potential (mV)
	1	2	3	4	5			
γ-Fe_2_O_3_						91	0.33	48
γ-Fe_2_O_3_@SiO_2_	SiO_2_					228 ± 1	0.16 ± 0.004	−54 ± 0.3
γ-Fe_2_O_3_@ SiO_2_-CS	SiO_2_	CS				226 ± 9	0.17 ± 0.02	7 ± 0.4
γ-Fe_2_O_3_@SiO_2_-CS-TA02	SiO_2_	CS	TA			212 ± 4	0.18 ± 0.01	−26 ± 1.2
γ-Fe_2_O_3_@SiO_2_-CS-TA10	SiO_2_	CS	TA			218 ± 2	0.16 ± 0.01	−21 ± 1.3
γ-Fe_2_O_3_@SiO_2_-CS-TA-CS	SiO_2_	CS	TA	CS		237 ± 12	0.19 ± 0.05	7 ± 0.2
γ-Fe_2_O_3_@SiO_2_-CS-TA10/2	SiO_2_	CS	TA	CS	TA	248 ± 8	0.17 ± 0.01	−20 ± 0.7
γ-Fe_2_O_3_@SiO_2_-NH_2_	SiO_2_	SiO_2_-NH_2_				246 ± 2	0.16 ± 0.01	39 ± 1.2
γ-Fe_2_O_3_@SiO_2_-NH_2_-TA02	SiO_2_	SiO_2_-NH_2_	TA			248 ± 14	0.18 ± 0.05	−21 ± 1.3
γ-Fe_2_O_3_@SiO_2_-NH_2_-TA10	SiO_2_	SiO_2_-NH_2_	TA			226 ± 6	0.18 ± 0.08	−25 ± 3.5

CS—chitosan, TA—tannic acid, *D*_h_—hydrodynamic diameter (DLS), *PD*—polydispersity (DLS).

## Data Availability

Data presented in this article is available on request from the corresponding author.
